# Social restructuring during harsh environmental conditions promotes cooperative behaviour in a songbird

**DOI:** 10.1098/rspb.2023.2427

**Published:** 2024-04-17

**Authors:** Ettore Camerlenghi, Sergio Nolazco, Damien R. Farine, Robert D. Magrath, Anne Peters

**Affiliations:** ^1^ School of Biological Sciences, Monash University, Wellington Road, Clayton, Victoria, Australia; ^2^ Department of Evolutionary Biology and Environmental Studies, University of Zurich, 8057 Zürich, Switzerland; ^3^ Department of Collective Behavior, Max Planck Institute of Animal Behavior, 78464 Konstanz, Germany; ^4^ Division of Ecology and Evolution, Research School of Biology, Australian National University, 46 Sullivan's Creek Road, Canberra 2600, Australia; ^5^ Department of Behavioural Ecology, Bielefeld University, 33615 Bielefeld, Germany

**Keywords:** cooperation, multilevel societies, superb fairy-wren, songbirds

## Abstract

Cooperation may emerge from intrinsic factors such as social structure and extrinsic factors such as environmental conditions. Although these factors might reinforce or counteract each other, their interaction remains unexplored in animal populations. Studies on multilevel societies suggest a link between social structure, environmental conditions and individual investment in cooperative behaviours. These societies exhibit flexible social configurations, with stable groups that overlap and associate hierarchically. Structure can be seasonal, with upper-level units appearing only during specific seasons, and lower-level units persisting year-round. This offers an opportunity to investigate how cooperation relates to social structure and environmental conditions. Here, we study the seasonal multilevel society of superb fairy-wrens (*Malurus cyaneus*), observing individual responses to experimental playback of conspecific distress calls. Individuals engaged more in helping behaviour and less in aggressive/territorial song during the harsher non-breeding season compared to the breeding season. The increase in cooperation was greater for breeding group members than for members of the same community, the upper social unit, comprised of distinct breeding groups in association. Results suggest that the interaction between social structure and environmental conditions drives the seasonal switch in cooperation, supporting the hypothesis that multilevel societies can emerge to increase cooperation during harsh environmental conditions.

## 1. Introduction

Cooperation is a crucial aspect of sociality, yet our understanding of what drives cooperative behaviour remains incomplete [1,2]. This is due to the complex interactions between facilitating factors and ultimate drivers. Specifically, while environmental conditions represent a strong candidate to drive the emergence of cooperation in animal societies, social structure is also likely to play a facilitating role [2]. In our study, we consider both factors and their interplay.

The structure of a population is predicted to have a strong influence on the evolution and maintenance of cooperative behaviour, because cooperation is thought to emerge through stable and repeated interactions among individuals [3–5]. For example, social network structures in primates, social insects and birds affect how individuals interact and also how they cooperate [6–8], with cooperation more likely to emerge in stable and well-structured social networks exhibiting high degrees of modularity [3]. Similarly, in hunter–gatherer societies, cooperation between groups is expressed more strongly when social structure is more stable [9], supporting the hypothesis that social structure facilitates the emergence of cooperation.

The expression of cooperative behaviour can also be driven by harsh environmental conditions (long-lasting conditions that threaten energy balance and survival). Theoretical studies suggest that such conditions favour cooperation because cooperating reduces the uncertainty experienced by individuals [10]. Mathematical models simulating human social structure and life history suggest that harsh and unpredictable conditions favour the emergence of cooperative behaviour [11] and that only cooperatively foraging phenotypes are likely to survive the harshest environmental conditions [12]. Recent evidence supports this hypothesis, showing that cooperation is often linked to environmental adversity in humans, primates and birds [13–16]. For example, in Diana monkeys between-group tolerance increases when conditions are physiologically stressful (i.e. low resources and high predation risk [15]), and increased predation risk enhances cooperation among breeding pied flycatchers *Ficedula hypoleuca* [16]. Furthermore, alloparental care in birds, humans and other mammals is associated with unproductive and unpredictable environments with low rainfall [13,14,17,18]. The benefits linked to cooperation under harsh conditions could then promote structured associations among individuals as they increase mutual tolerance and develop social bonds to gain benefits from reciprocal cooperative behaviours [2].

Studies on multilevel societies suggest links between social structure, environmental conditions and individual investment in cooperative behaviours [19]. Multilevel societies are formed when stable groups of individuals spatially and temporally overlap and associate preferentially with other groups, producing a nested social organization [20]. These societies are thought to provide individuals with access to a wider array of cooperative relationships, going beyond their immediate ‘strongly bonded' relationships, to social connections expressed more rarely. Evidence stems from two phylogenetically distant species, human hunter–gatherers and songbirds, where costs/risks associated with cooperative behaviours exhibit a hierarchical pattern that matches the multilevel structure of their societies [19,21]. In both societies, individuals invest in more costly cooperative behaviours towards members of their own core social unit, while restricting their cooperation to less costly behaviours when assisting individuals from the upper social unit to whom they are less strongly bonded [19,21]. This pattern suggests that multilevel social structures facilitate a larger pool of help in cooperative behaviours than would be expressed in a more unstructured society, where intermediate nested social levels are lacking. Furthermore, by reducing intergroup conflicts and increasing intergroup tolerance, multilevel societies might allow individuals to exploit larger areas during the harsher period of the year when fewer resources are available and individual mortality increases [22]. This is especially important when fewer resources are available, they are costlier to defend, and individual mortality increases [22]. For example, in southwest Africa and in Australia, where water availability often is asynchronous across distances of kilometres, tolerant relationships between indigenous hunter–gatherer communities become an important source of water access in case of local drought [23]. However, how environmental conditions and multilevel social structures interact to favour cooperative behaviours is difficult to test in wild populations.

Superb fairy-wrens (*Malurus cyaneus*) provide an ideal system for investigating the interaction between social structure, environmental conditions and cooperative behaviour. These songbirds live in a multilevel society comprised of distinct breeding groups that join communities with preferred neighbouring breeding groups and maintain the same membership to these communities across years [22]. The multilevel social structure of superb fairy-wrens emerges during the non-breeding season, the harsher (colder and less productive) time of the year, while during the breeding season each breeding group forms a territory that it defends from other groups [22]. Therefore, while communities form and dissolve, breeding group membership does not change across seasons, thus allowing us to test how variation in environmental conditions and in social structure predict individual investment in cooperative relationships.

To test the effect of social structure and environmental conditions on cooperation, we conducted playback experiments to stimulate individuals to express cooperative behaviour. We played distress calls from members of the breeding group and of the community, during the breeding and non-breeding season, and recorded all cooperative and aggressive territorial behaviours. This experimental design allowed us to test whether these behaviours vary with changes in (1) social structure, (2) environmental conditions or (3) the interaction between these two. If social structure determines cooperation (hypothesis 1), we predict increased cooperative behaviour and reduced aggressive territorial behaviour (territorial song) toward individuals from the same community during the non-breeding season, as community-level social interactions are present only during this time; we predict no change in behaviour towards members of the same breeding group, because these groups are stable year-round. If environmental conditions drive helping behaviour (hypothesis 2), we predict an overall increase in cooperation towards both community members and breeding group members and a reduction in song during harsher (non-breeding) conditions when survival benefits of cooperation are most important, particularly during the harshest weeks of the non-breeding season. If social structure and environmental condition interact to drive the expression of cooperative social behaviour (hypothesis 3), we expect to find individual responses to distress call playback to be more cooperative, and less aggressive and territorial, during non-breeding compared to breeding, with the magnitude of change in behaviour varying between the two social levels (breeding group, community). We specifically predict a greater increase in cooperative responses towards members of the breeding group, as individuals strongly benefit from the survival of other group members [24]. Finally, the null hypothesis is that individuals do not seasonally modulate their behaviour towards conspecifics, despite changes in social structure and environmental conditions.

## 2. Methods

### (a) Field study system

We collected data from a colour-banded population of superb fairy-wrens, a small (9–12 g) facultative cooperatively breeding songbird, at Lysterfield Park reserve, located near Melbourne, Australia (−37° 56′ 56.40″ S, 145° 17′ 45.60″ E) on the traditional land of Bunurong Boon Wurrung and Wurundjeri Woi Wurrung peoples of the Eastern Kulin Nation. The study area is comprised of open woodland, including areas with dense shrubs and open grassland. All individuals in the study were colour banded with a unique combination of one numbered metal ring issued by the Australian Bird and Bat Banding Scheme (ABBBS), an anodized coloured metal ring and two, coloured, plastic rings (in the top positions).

### (b) Social networks

We constructed social networks by regularly observing group co-membership among colour-banded individuals in the population. From the field observations of individuals in groups, we calculated the association strength between each individual and all other individuals in the population. The association strength was defined as the number of observations of the individuals in the same flock divided by the number of distinct observations in the same group or apart (i.e. the simple ratio index [25]). We then partitioned relationship types among individuals according to previously defined thresholds that correspond to the different levels in the multilevel society: the breeding group and the community [22]. To test the hypothesis that variation in social structure and environmental conditions can drive variation in helping behaviour, we focused on playback trials on the core social unit (the breeding group) and on the community level, which is composed of individuals interacting in the social network but with social connections varying seasonally.

### (c) Environmental harshness: weekly mortality hazard

We define harshness as long-lasting environmental conditions which decrease energy availability and reduce survival [26]. In general, winter is the harshest season for superb fairy-wrens, with highest mortality rate [27], and this is when the higher social level emerges. Additionally, as a proxy for weekly variation in environmental harshness during winter, we calculated the predicted weekly mortality hazard for superb fairy-wrens using previously published research [27], which showed that in this species, weekly mortality hazard rate is elevated with higher temperature maxima in the preceding two weeks and lower minima in the current fortnight [27]. To estimate the weekly mortality hazard for each week of our study period, we summed (number of days with maximum temperature ≥11°C during 1–12 days prior to the current week) + (number of days with minimum temperature ≤2°C during 17–22 days prior to the current week) following table 1 in Lv *et al*. [27]. Daily temperature was recorded by the Scoresby research institute (−37.8710, 145.2561; http://www.bom.gov.au/climate/data/), situated 8.8 km from the study site.

**Table 1.** An overview of hypotheses proposed to explain cooperative behaviour, their specific predictions with respect to the response by individual superb fairy-wrens to distress call playback, and whether these were supported in this study.

hypothesis	predicted responses to distress call playback in superb fairy-wrens	supported?
(1) variation in cooperation depends on changes in social structure	increased helping behaviour and reduced aggression/competition toward individuals from different breeding groups (same community) during the non-breeding season (when communities form)	no
(2) variation in cooperation depends on changes in environmental harshness	Increased helping behaviour and reduced aggression/competition during the harsher (non-breeding) season, particularly during the harshest weeks	no
(3) cooperative behaviour is affected by social structure and environmental conditions in interaction	increased helping behaviour and reduced aggression/competition during the non-breeding season but the magnitude of the change in behaviour varies between individuals from the same and from different breeding groups (same community)	yes

### (d) Experimental design

We broadcast conspecific distress calls to fairy-wren groups during the breeding season and the non-breeding season, to test how cooperation varies with sociality and environmental harshness. Distress calls are given in conditions of great danger, when individuals are confronted with or captured by a predator [28]. These calls can solicit aid from other fairy-wrens, including approach, mobbing and distraction displays, which entail close approach, assuming a hunched posture, and scurrying back and forth like a running rodent (‘rodent-run' behaviour) [21,29]. Alternatively, distress calls can also trigger aggressive territorial behaviour, in the form of singing. The first set of playbacks took place in September 2019, at the beginning of the breeding season. This is when breeding groups establish breeding territories and when, in many bird species, aggressive territorial behaviour related to territory defence peaks [30]. Specifically, in superb fairy-wrens, this is the time of the year when young females are evicted from their natal territory [31,32]. The second set of playbacks took place in June 2020, in winter when birds are not breeding and breeding groups associate with other breeding groups to form stable communities, the higher-level social unit [22]. The third set of playbacks was performed in September 2020, again at the beginning of the breeding season.

Our playback used a paired design, with each focal individual receiving two playbacks representing the two levels of the multilevel society (*n* = 94 individuals), following methods in [21]. Specifically, we presented individuals in each breeding group (association strength about 1) with a distress call recording from an individual from the same core unit, and from a different core unit within the same community (i.e. an individual it had repeatedly been observed with during the previous month; association strength greater than 0.05 and less than 0.41). The playback was accompanied by the presentation of a model predator, a taxidermied laughing kookaburra (*Dacelo novaeguineae*; we always used the same exemplar) to simulate a predation event. This species is a predator of the superb fairy-wren [33], and there have been multiple predation attempts, with two successful, at our study site (E.C. 2019–2020, personal observation) [34]. Calls used for playback were from seven females and ten males across 14 breeding groups. The order of the distress call presentation (within the same breeding group or community) was alternated to control for order effects. Half the groups were tested with distress calls recorded from a female, the other half from a male. Only one distress call was played per trial, with the second delayed for at least 5 days, to reduce any carry-over effects.

### (e) Distress call recording

Individuals were captured with mist nets to band and to record any distress calls produced when being extracted from the net. Individuals were recorded at a distance of approximately 30 cm on a Tascam Dr-40x recorder sampling at 44.1 kHz and with 24-bit resolution, and their identity noted. The recordings were made throughout 2019 and January–April 2020.

### (f) Playback field methods

Playbacks were carried out at an experimental arena set up near focal birds. Before each trial, one observer (EC) followed a given foraging breeding group from a distance of 10–15 m and identified all members from their unique colour band combinations. After this, which took at least 10 min, if all the birds appeared to be undisturbed by the observer presence, and we could not detect signs of predators, we set up the experimental trial. The experimental set up was located close to natural cover. Two dead branches without leaves (1 m long × 0.5 m high × 0.4 m wide) were placed to provide natural perches. The first branch was positioned 1 m from the closest natural cover; the second branch was positioned on the same line at 2 m from the natural cover. On the same line, at 1 m from the second dead branch and at 3 m from the natural cover, we positioned the predator model (covered by a cloth) and a tweeter speaker (Scan Speak Discovery, D2608). The speaker was remotely connected via an amplifier (12 v Kemo) to a solid-state media player (Sony Walkman NWZ-E383) and controlled by an observer (EC) hidden at 10 m from the model. Two Panasonic HC-V800 M camcorders were placed at a distance of two meters from the predator model at different angles and were activated by the second observer (SN). After this, the second observer (SN) uncovered the predator model and hid at a distance of 15 m from the experimental set-up; this procedure lasted approximately 60 s. The playback volume was set at 90 dBA at 0.2 m from the sound source, measured with a level meter, to simulate amplitudes recorded from birds in the wild.

The playback was started once at least one member of the group was at a distance of 10–15 m from the model predator. Each trial lasted 180 s starting from when a bird emitted the first alarm call or moved toward the speaker. However, if during the first 240 s of playback the birds did not give alarm calls, approach the speaker, or react by approaching the area, the trial was stopped and the birds were considered not responsive. When the birds were responsive, the observer (EC) recorded the behaviour and the identity of all individuals approaching the speaker.

We recorded whether individuals did or did not respond to playback, and the type of response if they did respond from the video recordings of the responses and the audio recordings using VLC Media Player. The scorer was blinded to the playback treatment by renaming the video files after removing the first 20 s of the videos and the audio recordings (where information about the trial was provided) and randomizing their order prior to analysis. We recorded whether individuals approached the model predator by flying toward it and whether they engaged in the risky 'rodent-run' display. We also recorded the identity of the first individual to approach. We categorized cooperative behavioural responses to playbacks on a 4-level scale representing increasing levels of risk: 0, no response; 1, approach the scene to mob the predator (low risk); 2, approach within 3 m of the model predator to mob (medium risk); and 3, perform a ‘rodent run' distraction display (high risk). Finally, during the playback trials, we recorded whether individuals in the group responded to distress calls with singing behaviour. All songs recorded were identified as song type I, which is associated with territorial behaviour [35].

### (g) Statistical analysis

To analyse differences in cooperative response levels (0–3 representing increasing levels of risk) we used cumulative link mixed models (CLMMs; using the package ‘ordinal' [36] in R (v. 3.5.1) in the form of ordinal logistic regressions) (model 1). We included season (breeding season, non-breeding season), the social relationship between the focal individual and the individual calling in the playback (a variable with two categories: same breeding group, same community), their interaction and the year when the experiment was performed as fixed effects in the model.

To further explore if within-season variation in environmental harshness can explain variation in cooperative behaviour within a season, we performed a separate analysis of playback responses during the non-breeding season, the harshest time of the year, when individual mortality risk is higher. To do so, we used a CLMM (model 2) similar to the one described above, except that fixed effects in this model were weekly hazard rate due to climate harshness (see above), and the social relationship between the focal individual and the individual calling in the playback.

To analyse differences in aggressive territorial responses, we built a GLMM (model 3) with a Bernoulli response distribution and a logit link function where the presence or absence of a recorded singing behaviour from at least one focal group member during the playback trial was our dependent variable and the breeding group was our unit of analysis. Given that singing was only recorded during the breeding season, we limited our analysis to the breeding-season, and thus excluded the variable season from model 3.

In all models, we included date of the experiment trial, trial order (1, 2, 3), individual (caller ID) and breeding group ID as random effects.

## 3. Results

**Figure 1.**
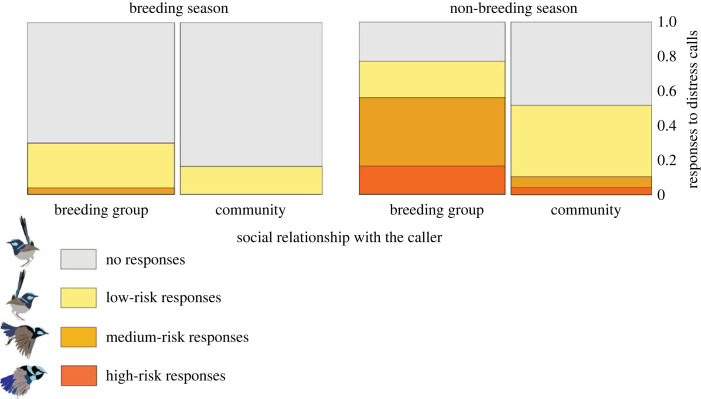
Seasonal changes in environmental conditions and social structure drive cooperative behaviours in superb fairy-wrens. During the non-breeding season, individuals increase the frequency and risk level of their helping behaviours towards breeding group members more than they increase towards individuals from neighbouring groups from the same community (interaction season × social relationship, *p* = 0.004), despite the fact that breeding groups remain unchanged across the seasons, whereas communities form and thus social bonds between community members increase in the non-breeding season. The *y*-axis represents the proportion of total responses to distress calls. Full details in table 2.

In all seasons, birds responded with more intense cooperative responses to distress calls from members of their breeding group than from other members of their community (social relationship between the caller and the focal bird is significantly positive *p* < 0.001; table 2). We also found a statistically significant interaction between the season and the social relationship with the caller (table 2; *p* = 0.004): birds increased their investment in helping behaviours in response to distress calls during the non-breeding season, and this seasonal increase was more marked towards members of their breeding group than towards other members of the same community (figure 1).

**Table 2.** Season and the social relationship with the caller interact to affect the intensity of superb fairy-wren responses to playback of distress call recordings. Shown is model output from a CLMM testing whether intensity (from 0 to 3) of responses to distress calls from conspecifics are explained by interaction between season (breeding versus non-breeding) and the hierarchical level of the social relationship with the caller (same breeding group, same community). Significant fixed terms are shown in italics; variance ± s.e. reported for random terms.

	predictor	effect ± s.e.	*p*-value
model	year2020	−0.22 ± 1.03	0.82
	time of day (scaled)	−0.02 ± 0.29	0.93
	*season (breeding)*^a^	*−2.7 ± 1.06*	*0.01*
	*social relationship with the caller (breeding group)*^b^	*2.88 ± 0.74*	*0.0001*
	*season × social relationship*	*−2.67 ± 0.92*	*0.004*
random terms	caller ID	6.25 ± 2.5	
	group ID	4.94 ± 2.22	
	date	1.47 ± 1.21	
	trial order	0.54 ± 0.73	

^a^‘Non-breeding season' is the reference term.

^b^‘Community level' is the reference term.

Birds exhibited stronger cooperative responses to distress calls during the non-breeding season compared to the breeding season (table 2). Additionally, they engaged in the most risk-prone helping behaviour, known as the ‘rodent run’, exclusively during the non-breeding season (figure 1). Although the weekly mortality hazard rate was significantly higher during the non-breeding season (ß = 1.07, s.e. = 0.27, *p* < 0.001), within the non-breeding season, weekly changes in mortality hazard were not associated with changes in superb fairy-wren helping or singing behaviour (table 3; *p* = 0.12).

**Table 3.** Weekly variation in mortality hazard due to environmental harshness season does not explain superb fairy-wren helping behaviour in response to playback of distress calls during the non-breeding. Shown is model output from a CLMM testing whether intensity (from 0 to 3) of responses to distress calls from conspecifics are explained by weekly mortality hazard rate due to environmental harshness (calculated from [27]); variance ± s.e. reported for random terms.

	predictor	effect ± s.e.	*p*-value
minimal model	weekly mortality hazard	0.96 ± 0.61	0.12
	time of day (scaled)	0.83 ± 0.55	0.13
random terms	group ID	9.49 ± 2.91	
	date	5.42 × 10^−12^ ± 2.39 × 10^−6^	
	caller ID	5.04 ± 2.24	
	trial order	4.17 ± 2.04	

**Figure 2.**
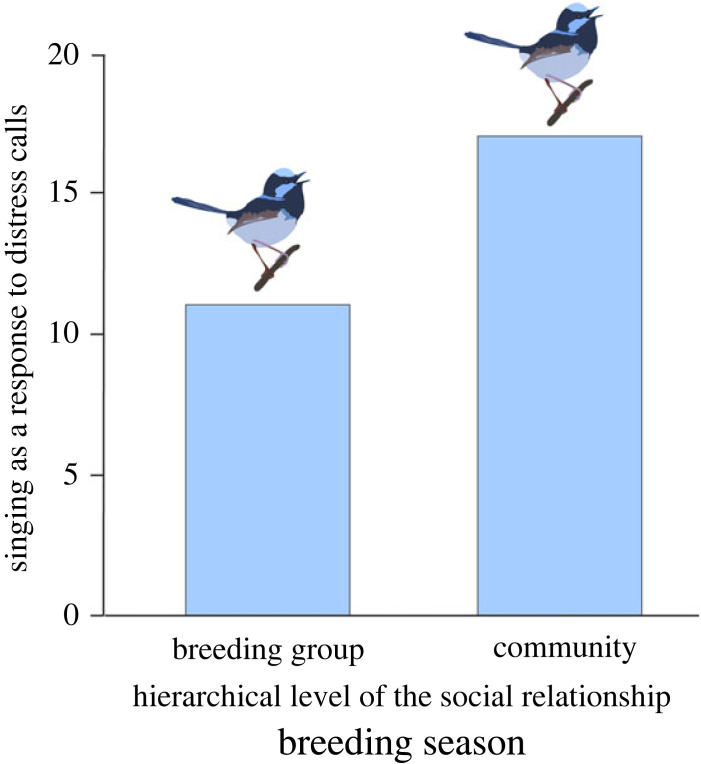
During the breeding season, fairy-wrens give fewer territorial songs in response to distress calls from breeding group members than members of the community. Individuals exhibit an increased territorial responses (singing) towards members of neighbouring groups (from the same community) compared to members of their own breeding group (*p* = 0.027); the singing response only occurs during the breeding season. The *y*-axis represents the number of playback trials in which a territorial response was recorded. Full details can be found in table 4.

Territorial responses (singing) occurred only during the breeding season, and birds sang more in response to distress calls from individuals within the same community compared to individuals from the same breeding group (figure 2 and table 4).

**Table 4.** Closer social relationship with the caller decreases the probability that superb fairy-wrens sing in response to distress call playback during the breeding season. Shown is model output from a GLMM testing whether probability of singing (yes/no) as a response to a distress call playback is predicted by the social relationship with the caller (same breeding group versus same community). No singing was recorded during the non-breeding season. Significant fixed terms shown in italics; variance ± s.e. reported for random terms.

	predictor	effect ± s.e.	*p­-*value
minimal model	*Year 2020*^a^	*−3.75 ± 1.66*	*0.024*
	time of day (scaled)	1.16 ± 0.71	0.11
	*social relationship with the caller (breeding group)*^b^	*−2.62 ± 1.18*	*0.027*
random terms	group ID	0.45 ± 0.67	
	date	0.61 ± 0.78	
	caller ID	0.00 ± 0.00	
	trial order	0.00 ± 0.00	

^a^Year 2019 is the reference term.

^b^Same community is the reference term.

## 4. Discussion

One of the major questions in biology is under which conditions cooperation emerges. In this study, we found that temporal variation in superb fairy-wren helping behaviour maps onto changes in social structure interacting with changes in environmental conditions (harshness). Our results therefore support the hypothesis that environmental conditions interact with social structure to drive variation in helping behaviour in superb fairy-wrens, and suggest that benefits linked to cooperation likely contribute to driving the seasonal restructuring in the multilevel society social structure.

Cooperation between individual superb fairy-wrens peaks during the winter months, outside the breeding season (table 2). By contrast, distress calls only elicit territorial song, which is an aggressive signal, during the breeding season (figure 2). While the latter might be related to reduced territoriality outside of the breeding season, this would not explain increased cooperative risk-taking behaviour. Similarly, while an increase in energetic constraints during the harsher months (e.g. due to food limitation, as in [37]) may (partly) explain the observed reduction in costly aggressive responses during winter, the high rate of energetically costly high-risk rodent-run behaviour displayed by superb fairy-wrens during winter does not support this hypothesis as fully explaining the observed changes in cooperation versus aggression. Instead, our results strongly align with previous studies suggesting that physiologically stressful environments and challenging conditions enhance the occurrence of cooperation [11,16]. The winter is harsher for insectivorous small birds, including superb fairy-wrens [38] and harsh conditions during this time reduce adult survival [27,39,40]. Individuals can benefit in a range of ways by being more cooperative during harsh winter conditions. For example, reduced inter-group aggression [41,42] facilitates discovery of food [43], allows individuals to exploit larger areas [22], and favours communal defence against predators [44]. Such benefits should be particularly enhanced in cooperatively breeding birds, as individuals benefit not only themselves, but also from the survival of other group members—both directly (group-living benefits) and indirectly (e.g. kin selection) [24,45]. Thus, our results support the idea that environmental harshness can contribute to the emergence of cooperation and corroborates recent findings suggesting that in many bird species, it is during winter months that complex social behaviour is likely to take place [22].

While fairy-wrens exhibit an increase in cooperation during the harshest season, it is more pronounced towards members of their breeding group than towards other members of the same community. This makes sense since cooperative anti-predator responses that enhance survival of breeding group members, that share close social relationships year-round and often are related, might bring greater direct and indirect benefits [45]. Likewise, during the breeding season, when breeding groups form territories, individuals increase their territorial (song) responses, particularly toward distress calls of members from the broader community rather than those in the same breeding group. As individual responses overall were thus more cooperative and less aggressive during the harsher season, with the magnitude of the behaviour change varying between the breeding group members and community members, our results support the hypothesis that cooperative behaviour is influenced by social structure and environmental conditions in interaction.

Our study suggests that harsher environmental conditions can lead to a reorganization of the social structure and an increase in cooperative behaviour. These two elements should not be considered independently, as they are likely to emerge in an iterative manner. In response to harsh environmental conditions, cooperation can emerge in the form of reduced aggression rates towards potential competitors that can increase interdependence among individuals (through group size benefits). Such interdependence would further drive the benefits of maintaining relatively stable associations with extra-group members, as repeated low-cost interactions could allow more expensive forms of cooperative behaviours to emerge [46,47]. Such a positive feedback loop between cooperation and social structure [2] could then drive the emergence of upper social units within multilevel societies.

Multilevel societies might represent a functional strategy to overcome the challenges posed by harsh conditions, offering an alternative solution to the approach based on redundancy, adopted by many eusocial species. Redundancy, where groups contain more members than strictly necessary to accomplish specific tasks, is often considered to enhance group resilience in harsh and uncertain conditions [48]. Ant colonies, for example, exhibit extensive redundancy, with a significant number of inactive workers that can be mobilized for group tasks such as foraging or caring for the brood as needed [49]. By contrast, multilevel societies, through fission–fusion dynamics between groups, provide the flexibility to adjust group size depending on changing environmental conditions and resource availability, eliminating the need for excessive redundancy, which can be costly. This process can occur in different ways. Firstly, groups may split into smaller units to reduce competition when the environment becomes more unpredictable, particularly in cases of limited and dispersed resources, as observed in Hamadryas baboons (*Papio hamadryas*) and African elephants (*Loxodonta africana*) [50,51]. Secondly, territorial species can increase intergroup tolerance, enabling individuals to exploit larger areas during harsh periods with limited resources [22]. Finally, in some species, during times of scarcity and resource limitations, groups may coalesce to engage in cooperative behaviours, providing protection against predators and sharing information about resource distribution. This phenomenon has been observed in vulturine guineafowl (*Acryllium vulturinum*) and killer whales (*Orcinus orca*) [52,53]. Hence, multilevel societies are likely to offer an efficient solution for social species to cope with environmental uncertainty and harshness.

Our work also raises new perspectives on the formation of non-breeding groups. Specifically, increased tolerance among individuals under harsher environmental conditions could be a key driver facilitating the emergence of multilevel social structure through interdependence. In birds, this generally coincides with non-breeding periods. However, not all birds form stable social units outside of the breeding season. For this reason, we encourage researchers to investigate cooperative and altruistic behaviours during the non-breeding season, which historically has received far less attention than the breeding period [52] and would contribute to better understanding how and when cooperative behaviours emerge in wild animal populations. Specifically, we believe that further studies investigating the directional link between social structure and cooperative behaviour might help to shed light on the conditions favouring the emergence of cooperative behaviour in wild animal societies.

## Data Availability

Data used for analysis can be accessed at Figshare [54].
